# Metastatic Malignant Melanoma of Parotid Gland with a Regressed Primary Tumor

**DOI:** 10.1155/2016/5393404

**Published:** 2016-07-05

**Authors:** M. Mustafa Kılıçkaya, Giray Aynali, Ali Murat Ceyhan, Metin Çiriş

**Affiliations:** ^1^School of Medicine, Otolaryngology Department, Suleyman Demirel University, 32260 Isparta, Turkey; ^2^School of Medicine, Dermatology Department, Suleyman Demirel University, 32260 Isparta, Turkey; ^3^School of Medicine, Pathology Department, Suleyman Demirel University, 32260 Isparta, Turkey

## Abstract

Malignant melanoma of the parotid gland is often metastatic and mainly originates from malignant melanomas in the head and neck. Nevertheless, some malignant melanomas may metastasize and subsequently regress. Therefore, it may not be possible to observe a metastatic malignant melanoma and its primary melanoma simultaneously. The investigation of a patient's old photographs may help in the detection of preexisting and regressed pigmented lesions in the facial and neck regions.

## 1. Introduction

Primary malignant tumors of the salivary gland are observed with a prevalence rate of one per 100,000 people. Generally, 80% of all salivary gland tumors develop in the parotid gland, and 25% of those are malignant. Adenoid cystic carcinoma, mucoepidermoid carcinoma, and acinar duct carcinoma are the most frequently observed types of salivary gland cancers [1]. The relevant literature reports widely varying rates of parotid gland tumors accompanied by facial nerve palsy. According to Stodulski et al., this rate is about 20.6% [2]. Moreover, Leverstein et al. report that half of the parotid gland tumors accompanied by facial nerve palsy are adenocarcinomas [3]. About 25% of parotid tumors are metastatic, and, to a large extent, they originate in the head and neck. Squamous cell carcinoma and malignant melanoma are the most common types of cancer with metastasis to the parotid gland [4].

## 2. Case Report

**Figure 1:**
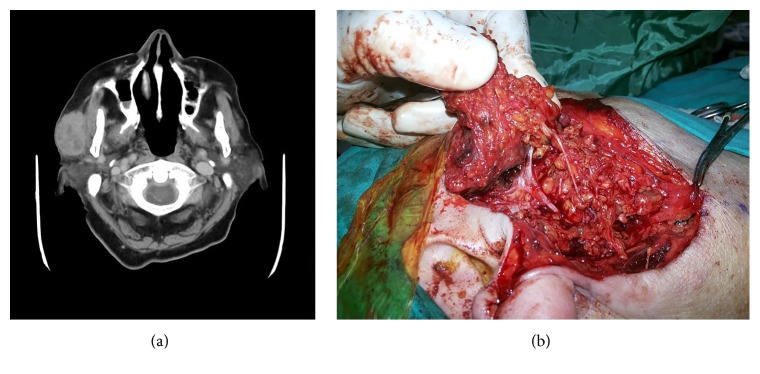
(a) Lobular-contoured solid mass in the anterior lobe of the right parotid gland (4 × 2.5 cm in size) in the patient's computed tomography (CT). (b) The lesion invading temporozygomatic and buccal branches of the facial nerve and originating from the superficial lobe of the parotid gland.

A 79-year-old female patient with a history of appendectomy and cholecystectomy was diagnosed with hypertension and diabetes mellitus. She was referred to an outpatient clinic with a complaint of a 3 × 4 cm mass that had been located in the right parotid region for eight months and recently grew rapidly to cause total facial nerve palsy. The mass was mobile, and the patient did not report pain. The patient's computed tomography (CT) indicated a lobular-contoured, 4 × 2.5 cm solid mass lesion with heterogeneous contrast enhancement in the anterior lobe of the right parotid gland (Figure 1(a)). A fine needle aspiration biopsy revealed a malignancy of an unknown type. Neither physical and endoscopic examinations nor positron emission tomography-computed tomography (PET-CT) checks succeeded in establishing the primary origin of the malignancy.

**Figure 2:**
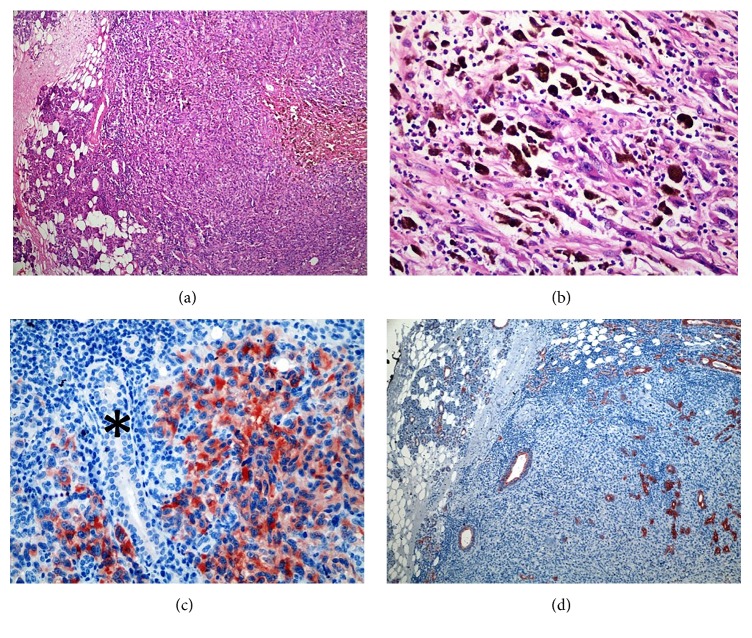
(a) Infiltrated parotid tumor. Normal parotid tissue in the left (residue) (100x HE). (b) The melanin-rich tumor (400x HE). (c) The analysis identified positive staining for HMB-45 (400x). Asterisk: salivary duct, negative staining for HMB-45. (d) Negative staining for PanCK of the tumor and positive staining for PanCK in the residue parotid tissue (PanCK, 100x).

However, an elevated skin flap revealed a pigmented lesion that had infiltrated the superficial parotid gland. Following a frozen-section examination, the patient was diagnosed with melanoma. We observed the temporozygomatic and buccal branches of the facial nerve penetrating a 5 × 6 cm mass originating from the superficial lobe of the parotid gland (Figure 1(b)). We then performed a total parotidectomy operation in which we resected the temporozygomatic and buccal branches and dissected the deep lobe. The results of an immunohistochemical analysis found the melanoma to be HMB-45 positive, S100 positive, Melan-A positive, and PanCK negative. These findings verified the malignant melanoma diagnosis (Figures 2(a), 2(b), 2(c), and 2(d)).

**Figure 3:**
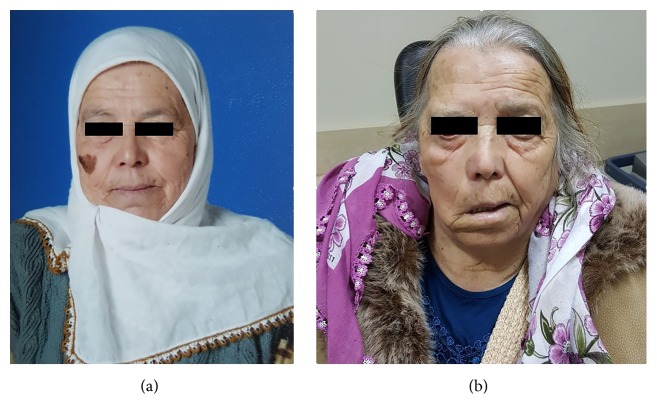
(a) 3 × 3 cm hyperpigmented lesion in the right facial region was detected in photographs that were taken five to six years ago. (b) The spontaneous regression of hyperpigmented lesion.

No primary origin of the melanoma was detected in the head, the neck, or any other region during the patient's postoperative dermatology consultation. Considering the possibility that the primary metastatic malignant melanoma might have regressed, we decided to check the patient's earlier photographs. Upon examination of photographs that were taken five to six years ago, we detected a 3 × 3 cm hyperpigmented lesion in the right facial region (Figures 3(a) and 3(b)). The patient reported that the lesion gradually disappeared spontaneously. We considered the possibility that the metastatic malignant melanoma's primary tumor was likely to have regressed at a later time.

## 3. Discussion

Primary malignant melanomas of the parotid gland are extremely rare. Some melanomas may exhibit no or low pigmentation, and these are called amelanotic melanomas. Malignant melanomas of the parotid gland are largely metastatic, with primaries usually originating in the skin of the head and neck [5]. About 10% to 35% of melanomas present regression and even disappear completely. Regressed melanomas can be categorized into two groups: lentigo maligna melanoma and superficial spreading melanoma. Some studies suggest that regressed malignant melanomas are more likely to metastasize [6]. The parotid tissue is an uncommon location for metastases; it may involve head and neck carcinomas and cutaneous melanomas. More than 75% of parotid metastases are believed to spread into the intraparotid lymph nodes of a nearby malignancy. The parotid gland is known to function as a filtering center for lymphatic drainage in the head and neck region [7]. Some studies have reported facial melanomas with metastasis to the parotis and ipsilateral levels I–IV [8].

In our case, when the patient was diagnosed with a malignant parotid melanoma, we detected neither a pigmented lesion nor an amelanotic lesion to suggest a primary tumor in the head, the neck, or any other body part. The patient's earlier photographs revealed a pigmented lesion on the ipsilateral facial skin, which later disappeared spontaneously. As no other primary tumor was found to account for the metastasis, we considered the previous lesion to be a regressed malignant melanoma with metastasis to the parotid.

Some studies recommend superficial parotidectomy and radiotherapy for superficial lobe involvement in the case of metastatic parotid cancers, whereas others recommend total parotidectomy and neck dissection given the possibility of an occult invasion in the deep lobe in the case of superficial lobe metastasis [9, 10]. Other studies report facial nerve palsy in approximately 20% of parotid malignancy cases [2]. In our case, the facial nerve was invaded, and we therefore performed a total parotidectomy.

In conclusion, it may not be possible to observe metastatic parotid malignant melanoma and its primary melanoma simultaneously. For this reason, it may be helpful to check earlier photographs of patients to detect a possible regressed malignant melanoma.
